# Subthalamic Nucleus Oscillatory Characteristics in Meige, Cervical Dystonia and Generalized Dystonia

**DOI:** 10.1002/acn3.70040

**Published:** 2025-04-03

**Authors:** Zhu Guan‐Yu, Yin Zi‐Xiao, Chen Ying‐Chuan, Timon Merk, Thomas Binns, Ma Ruo‐Yu, Du Ting‐Ting, Liu Yu‐Ye, Xie Hu‐Tao, Shi Lin, Yang An‐Chao, Meng Fan‐Gang, Wolf‐Julian Neumann, Andrea A. Kühn, Zhang Jian‐Guo

**Affiliations:** ^1^ Department of Neurosurgery Beijing Tiantan Hospital, Capital Medical University Beijing China; ^2^ Movement Disorder and Neuromodulation Unit, Department of Neurology Charité—Universitätsmedizin Berlin Germany; ^3^ Department of Functional Neurosurgery Beijing Neurosurgical Institute, Capital Medical University Beijing China; ^4^ Beijing Key Laboratory of Neurostimulation Beijing China

**Keywords:** dystonia, local field potentials (LFPs), subthalamic nucleus (STN)

## Abstract

**Objective:**

Deep brain stimulation offers a unique opportunity to record neural activity of the basal ganglia. While much work in dystonia has focused on the globus pallidus internus, expanding research to investigate subthalamic nucleus (STN) activity in various dystonia types is critical to provide a comprehensive understanding of dystonia pathophysiology.

**Methods:**

STN and cortex activity were recorded from 17 patients with cervical dystonia (CD), 19 with Meige syndrome, and 9 with generalized dystonia (GD) during the lead externalized period. We investigated local and network oscillatory characteristics, including power, bursts, and coherence. Additionally, we explored the relationship between these features and the severity of dystonic symptoms within each group and conducted a comparative analysis across the different dystonia types.

**Results:**

Peaks of low‐frequency (4–13 Hz) and beta (14–30 Hz) power were present in the STN of all patients; most of the beta peaks are distributed in the high beta range (20–30 Hz). The CD and GD groups showed longer low‐frequency bursts and greater high beta power in STN than the Meige group. Interestingly, the CD group showed stronger STN‐cortex low‐frequency coherence, while the GD group had stronger STN‐cortex high beta coherence. Combined, low‐frequency and beta features could predict symptom severity with a performance of 73% in the CD group and 82% in the GD group.

**Interpretation:**

Low‐frequency and high‐beta oscillations are present in the STN across all three types of dystonia. The distinct patterns may be associated with different underlying pathological mechanisms.

## 1 Introduction

Dystonia is a neurological movement disorder characterized by involuntary muscle contractions, which can lead to repetitive movements, abnormal postures, and significant discomfort [1]. Deep brain stimulation (DBS) has emerged as a compelling therapeutic option for patients with severe isolated idiopathic dystonia who exhibit limited responsiveness to other interventions [2]. The advent of DBS also provided a unique window of research opportunity. During surgical implantation and in the subsequent postoperative period with externalized electrodes, DBS allows for the direct recording of oscillatory activity within the basal ganglia [3]. In dystonia, several studies have highlighted low‐frequency activity (4–13 Hz) as a potential biomarker associated with dystonia severity; however, most work has focused on the activity of the globus pallidus internus (GPi) [4, 5, 6]. Recently, the subthalamic nucleus (STN) has emerged as a compelling target for DBS in dystonia, rivaling the established role of the GPi. While studies have demonstrated comparable therapeutic efficacy of DBS between these two targets, the STN offers advantages in terms of targeting accuracy and reduced battery consumption, making it a promising option for future DBS applications in dystonia [7]. Yet, given the few investigations of STN oscillatory activity in dystonia patients, there exists a limited understanding of dystonic biomarkers in the STN.

Among the diverse types of dystonia, Meige syndrome, cervical dystonia (CD), and generalized dystonia (GD) represent distinct clinical presentations with unique features and challenges. Understanding the electrophysiological characteristics of these disorders will be crucial for developing effective treatment strategies and providing comprehensive care for individuals affected by these challenging conditions. Indeed, the GPi and STN exhibit distinct neural circuitry, with the STN receiving direct, monosynaptic connections from the cortex in the form of the hyperdirect pathway. While this study does not directly investigate the hyperdirect pathway itself, we aim to explore the interactions between the cortex and STN, which are anatomically supported by this pathway. Indeed, a recent study found that STN‐DBS directly modulates the activity of the cortex, an effect not seen for GPi‐DBS [8]. Therefore, investigating this interaction in different kinds of dystonia could be of special interest as abnormal cortical activity is another major pathophysiological hallmark of dystonia.

To this end, we recorded simultaneous STN and cortex activity in 45 dystonia patients with idiopathic Meige syndrome, CD, and GD receiving STN‐DBS. The following questions were investigated: (1) STN‐local field potentials (LFPs) characteristics—How do oscillatory characteristics of the STN compare among these dystonia subtypes? (2) Cortex‐STN interactions—Do coupling between the cortex and STN differ between the dystonia subtypes? (3) LFPs features and dystonia severity prediction—Do oscillatory features predict the severity of each dystonia subtype?

## 2 Patients and Methods

### 2.1 Patients and Surgery

We focused on patients with idiopathic isolated dystonia to obtain neurophysiological data from a homogenous cohort of dystonia patients without clear etiology. We recruited 45 patients (22 female, age: 48.6 ± 15.0 years, disease duration: 6.0 ± 4.8 years) diagnosed with Meige syndrome, CD, and GD. A detailed clinical examination was performed to exclude secondary neurological conditions and rule out abnormalities on MR imaging. Standard blood tests assessed overall health, with additional copper and ceruloplasmin testing performed when necessary to exclude Wilson's disease. Only patients without known genetic mutations associated with dystonia were included (TOR1A, TAF1, GCH1, SPR, TH, THAP1, PNKD, SGCE, DRD2, ATP1A3, SLC2A1, PRRT2, PRKRA, GNAL). The study was approved by the local ethics committee and adhered to the standards set by the Declaration of Helsinki. Written informed consent was obtained from all patients before their inclusion in the study. CD symptom severity was assessed using the severity subscale of the Toronto Western Spasmodic Torticollis Rating Scale (TWSTRS); Meige and GD syndrome severity were assessed using the motor subscale of the Burke‐Fahn‐Marsden Dystonia Rating Scale (BFMDRS) by an experienced movement disorders specialist as part of the clinical routine. The detailed information is shown in Table S1.

All patients underwent bilateral STN electrodes implantation. Electrodes were aimed at the dorsolateral part of the STN, targeting the basal ganglia motor circuit using the SurgiPlan system (ELEKTA, Stockholm, Sweden). Microelectrode recordings were performed for precise localization of the STN, at which point the macroelectrodes were implanted. PINS L301 (PINS, Beijing, China) or Medtronic 3389 (Medtronic, Minneapolis, USA) leads were implanted into the STN; the four contacts had a contact length of 1.5 mm and an equal distance of 0.5 mm to another. The diameter of the contacts was 1.27 mm. To maintain consistent terminology, we have adopted a uniform numbering system (the caudal bipolar contact pairs is 1–2). Electrode contact positions were confirmed through the fused postoperative CT with preoperative MRI. DBS electrodes were visualized using the advanced processing pipeline in Lead‐DBS [9].

### 2.2 Experiments and Recordings

Subjects underwent recordings during lead externalization, which began 2–4 days after surgery. For analysis, data were selected from periods when patients remained at rest, lying comfortably in bed with instructions to avoid voluntary movements and to keep their eyes open. Anti‐dystonia medication was discontinued at least 12 h on the day of recording. None of the patients had received botulinum toxin injections within the 6 months prior to the recording period. Surface electroencephalogram (EEG) was recorded (frontal: F3, F4; central: C3, C4, as per the 10–20 system). Subthalamic LFPs were recorded bipolarly from adjacent contacts (1–2, 2–3, and 3–4) using the JE‐212 amplifier (Nihon Kohden, Tokyo, Japan). The amplification gains and hardware filter settings were standardized at ×195 and within the range of 0.08–300 Hz, respectively. Signals were digitized at a sampling rate of 1000 or 2000 Hz.

### 2.3 Power and Location Analysis

Offline signal analysis was performed using MNE‐Python [10], SciPy [11], and py_neuromodulation [12]. 5 min of artifact‐free data were selected for analysis. To minimize selection bias, all possible contact pairs were included in the analysis. Signals were downsampled to a common sampling rate of 1000 Hz. Spectral analysis included a total of 360 subthalamic LFP contact pairs from 90 dB electrodes implanted in 45 patients. Additionally, 90 EEG contact pairs (F3‐C3 or F4‐C4) in the same 45 patients were analyzed. All continuous data were epoched into arbitrary segments of 1.024 s length (1024 samples). Power spectral densities of the epoched data were computed using Welch's periodogram with a fast Fourier transform method. Power was normalized to the standard deviation of the 4–45 Hz and 55–95 Hz bands as described previously [13]. Power was averaged within the normalized spectra across the following four canonical frequency bands: theta (4–8 Hz); alpha (9–13 Hz); low‐frequency (4–13 Hz); low beta (14–20 Hz); high beta (21–30 Hz); beta (14–30 Hz). We used the find_peaks algorithm in sciPy and visually checked the spectral peaks for descriptive statistics. In contacts where more than one peak was found in each frequency band, we chose the peak with the highest prominence for further analysis. For each electrode, peak amplitude across all channels was averaged. For each subject, bilateral peak amplitude was averaged to get one value for clinical score correlation.

To investigate the low‐frequency and high beta power distribution within STN using electrode localizations, the recorded power values were mapped into standard stereotactic MNI space following the subcortical electrophysiology mapping methodology [14]. All patients' normalized band power was assigned to the Euclidean midpoint between the constituent contacts of the bipolar contact pairs. Data from the left hemisphere were mirrored onto the right hemisphere after verification that there were no significant differences in power between hemispheres. Frequency band power within the standard space was interpolated to the STN surface using natural neighbor interpolation and smoothed with a Gaussian kernel using a full‐width half‐maximum of 0.7 mm. Locations of greatest 5% power in each frequency band were determined by average coordinates across three axes.

### 2.4 Coherence and Burst Analysis

Coherence analysis between the STN and ipsilateral EEG was performed to investigate their functional connectivity. Coherence provides a frequency domain measure of correlation between signals, revealing spectrally specific functional connectivity between neuronal populations. For the coherence analysis, spectral information was again computed using Welch's method. Coherence of the original data was compared to coherence from surrogate data in which the phase alignment of signals was shuffled. The coherence in the peak located frequency band (low‐frequency: 4–13 Hz; high beta: 20–30 Hz) was averaged for all contact combinations between bipolar STN channels and ipsilateral F3‐C3 or F4‐C4, given their locations in motor and premotor areas. For each subject, STN‐cortex coherence from each hemisphere was averaged to produce a single connectivity value for clinical score correlation.

For burst analysis, the signal was DC corrected, smoothed, and rectified. The amplitude threshold was set to 75%, as utilized in previous reports [15]. Burst analysis was performed using the py_neuromodulation toolbox [12], and average burst length was computed.

### 2.5 Feature Extraction and Clinical Score Prediction

For each subject, we extracted three subthalamic band power features (low‐frequency (4–13 Hz); low beta (14–20 Hz); high beta (21–30 Hz)), two cortex‐STN band coherence features (low‐frequency: 4–13 Hz; high beta: 20–30 Hz), two averaged burst length features (low‐frequency: 4–13 Hz; high beta: 20–30 Hz), and peak amplitude (low‐frequency: 4–13 Hz; beta: 14–30 Hz), resulting in a total of 9 features per subject. All features were extracted by averaging the feature in each contact per electrode, and further averaged across hemispheres. We performed a minimum‐redundancy maximum‐relevance (mRMR) feature selection [16]. The mRMR algorithm operates through an iterative optimization process that maximizes feature‐target relevance while minimizing inter‐feature redundancy. The selection of two features was driven by the small sample size, which necessitated limiting features to mitigate overfitting risks and ensure robust validation. The selected features were then subjected to the linear regression model. Our preference for the above process stems from two key considerations. First, more complex models exhibit a greater propensity to overfit the data, leading to poor generalizability [17]. Second, beyond achieving a strong correlation, we prioritized gaining insights into the underlying mechanisms that link these features to the severity of clinical symptoms. Model performance was evaluated using leave‐one‐subject‐out cross‐validation.

### 2.6 Statistical Analysis

All statistical analyses were conducted using SciPy [11]. The nonparametric Wilcoxon signed‐rank test, Mann–Whitney *U* test, and Kruskal–Wallis test were used wherever applicable. For correlation analyses, both Spearman's rho and Pearson's *r* were reported to account for potential influence by extreme values. Multiple comparisons were controlled using the Bonferroni correction. *p*‐values less than 0.05 were considered statistically significant.

## 3 Results

### 3.1 Peak Characteristics Across Patients and Subtypes

**FIGURE 1:**
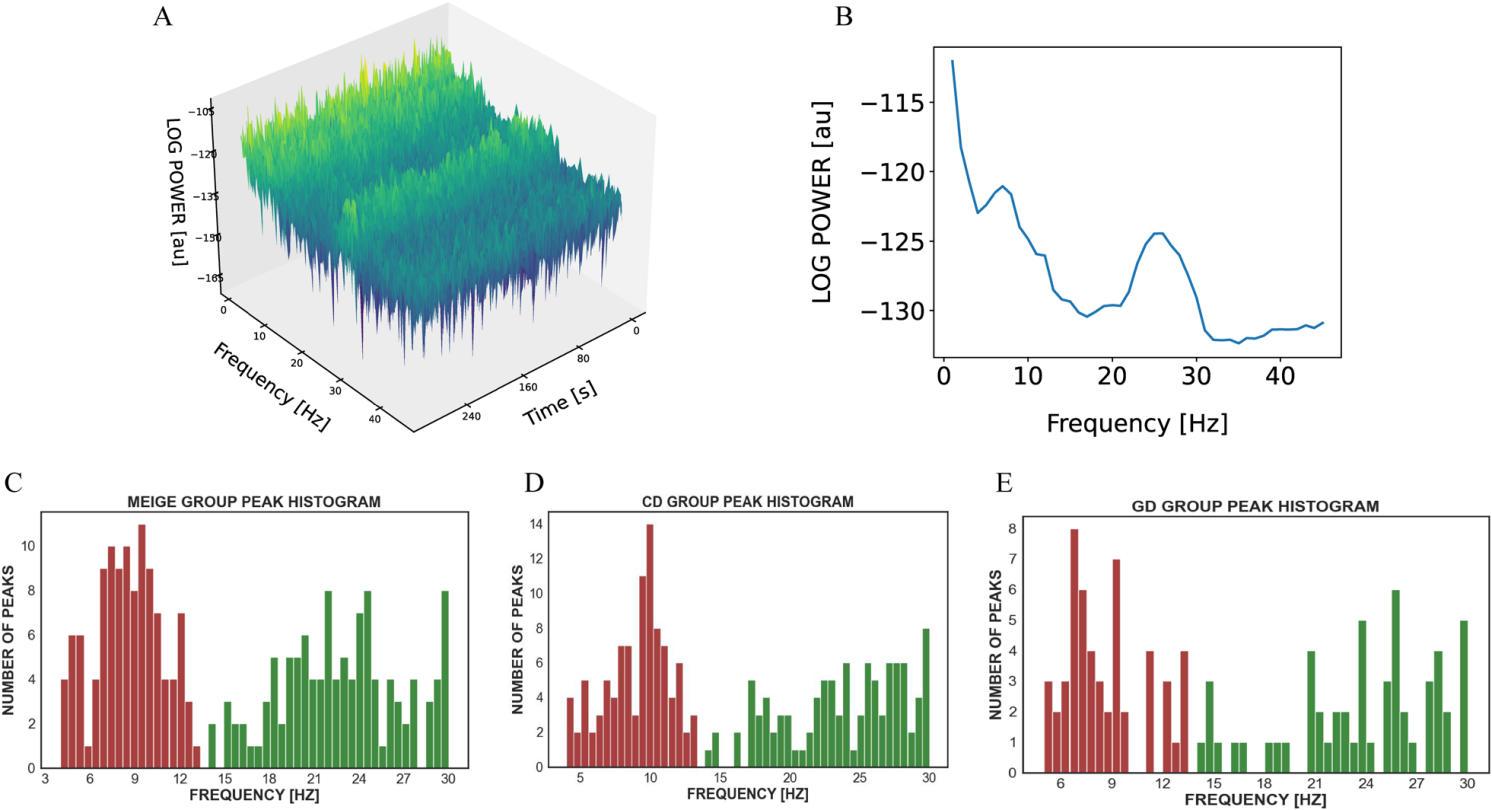
Subthalamic nucleus oscillatory activity. (A) An example patient from the CD group (CD #12), with a time‐frequency plot showing the power peaks at 7 Hz and 26 Hz, and (B) further averaged over time. (C–E) Peak histogram of MEIGE, CD, and GD, respectively. low‐frequency (4–13 Hz) peak (red), beta (14–30 Hz) peak (green). All local field potentials were recorded in a bipolar manner from adjacent contact pairs. Beta peaks were located primarily in the high beta band (21–30 Hz; Meige: 88 out of 114 pairs; CD: 80 out of 102 pairs; GD: 44 out of 54 pairs). The low‐frequency and beta peak distributions showed no significant differences between the three groups (Kruskal–Wallis test, *p* > 0.05). CD, cervical dystonia; GD, generalized dystonia.

Patients across all dystonia types exhibited peaks of power in the low‐frequency (4–13 Hz) and beta (14–30 Hz) bands in STN (Figure 1A,B). Analysis of peak power amplitudes revealed an average decrease of 26.4% ± 3.4% from the maximum point to other contact pairs, suggesting a localized source. Additionally, the average difference in peak frequency between maximum and other contacts was minimal (0.36 ± 0.33 Hz), further supporting a focal origin. Most of the beta peak distributed in the high beta frequency range; the low‐frequency and beta peak distributions showed no significant differences between the three groups (Kruskal–Wallis test, *p* > 0.05) (Figure 1C–E).

### 3.2 Power Spectrum and Burst Length Comparison

**FIGURE 2:**
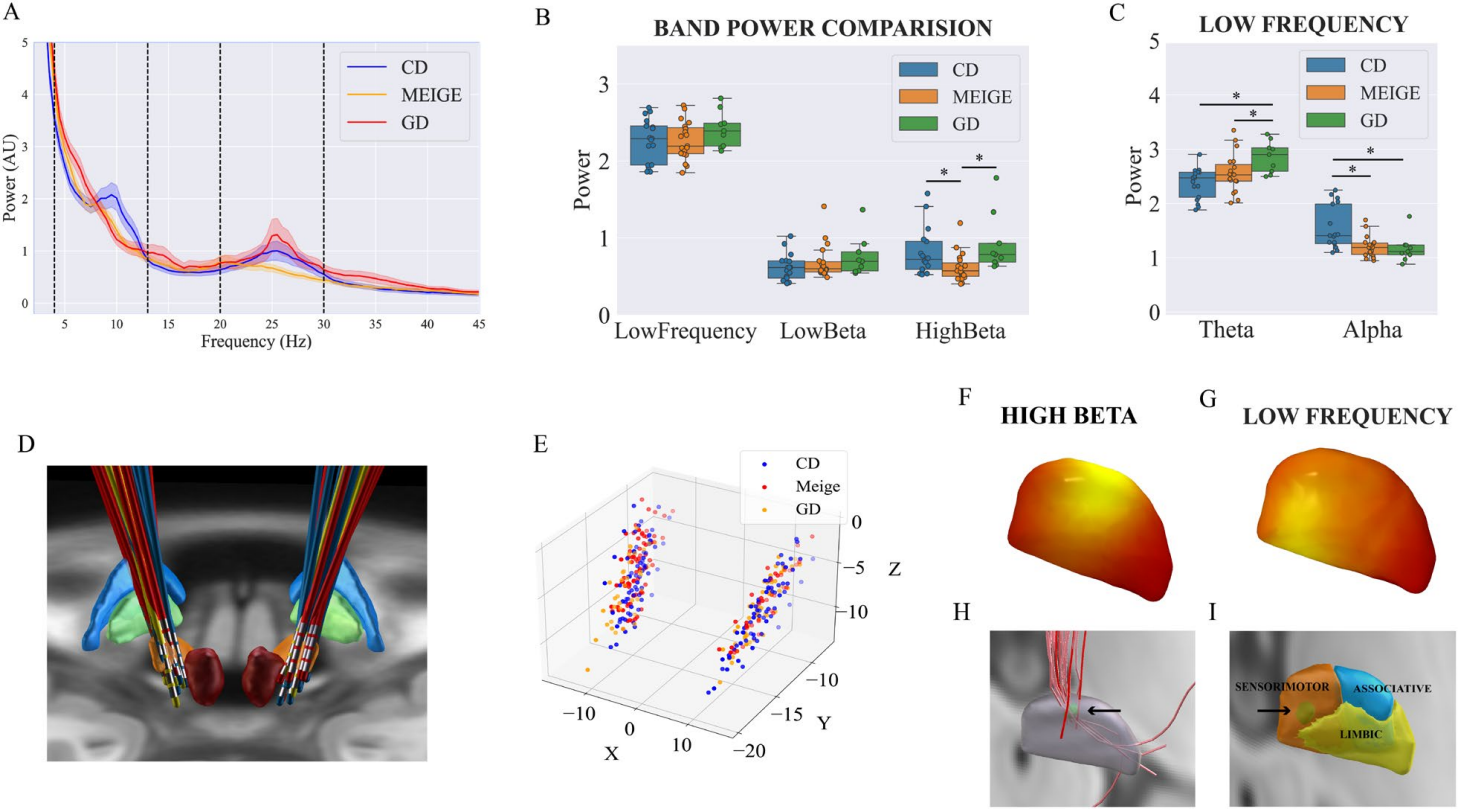
Power spectra comparison and spatial distributions. (A) Power spectrum for CD, GD, and Meige groups (Mean and S.E.M), with dashed lines separating frequency bands at 4, 13, 20, and 30 Hz. (B) Average band power comparison revealed a significant difference in high beta range, with greater high beta power in the CD and GD groups compared to the Meige group (Kruskal–Wallis test, *p_Bonferroni_ < 0.05). (C) In the low frequency range, there was larger theta power in the GD group and larger alpha power in the CD group compared with the other two groups (Kruskal–Wallis test, *p_Bonferroni_ < 0.05). (D) DBS lead location reconstructions. (E) Contact locations in MNI space, with no significant differences in locations between groups (Kruskal–Wallis test, *p* > 0.05). (F) High beta power spatial distribution in STN. (G) Low‐frequency power spatial distribution in STN. (H) Locations of top 5% high beta power location overlap with hyperdirect pathway fibers in STN (green sphere). (I) Locations of top 5% low‐frequency power location overlap with the ventral portion of sensorimotor STN (green sphere). Low‐frequency power is also mapped to the STN surface. CD, cervical dystonia; GD, generalized dystonia.

The power spectrum for each dystonia subtype (CD, Meige, and GD) is illustrated (Figure 2A). Notably, both the GD and CD groups exhibited significantly greater high beta band power compared to the Meige group (*p* < 0.05) (Figure 2B). In the low frequency band range, the CD group has significantly higher 8–13 Hz power and the GD group has significantly higher 4–8 Hz power compared to the other two groups (*p* < 0.05) (Figure 2C). No significant differences in STN electrode locations were found between the groups (Figure 2D,E), indicating that the power differences were caused by the disease type rather than the electrode location.

Analysis of the spatial distribution of power in the STN revealed a clear topographical distinction: high beta power was strongest in the dorsal region of the STN, while low‐frequency power was strongest ventrally (Figure 2F,G). To further investigate these spatial patterns, we examined the MNI coordinates of the top 5% high beta power locations (*X*: ±12.78 mm, *Y*: −12.41 mm, *Z*: −5.78 mm). Interestingly, these coordinates closely align with the distribution of hyperdirect fibers, suggesting a link between high beta activity and this neural pathway. The top 5% low‐frequency power locations (*X*: ±13.68 mm, *Y*: −15.03 mm, *Z*: −7.36 mm) were located within the ventral motor region (Figure 2H,I), potentially reflecting the role of low‐frequency activity in motor control processes interference.

**FIGURE 3:**
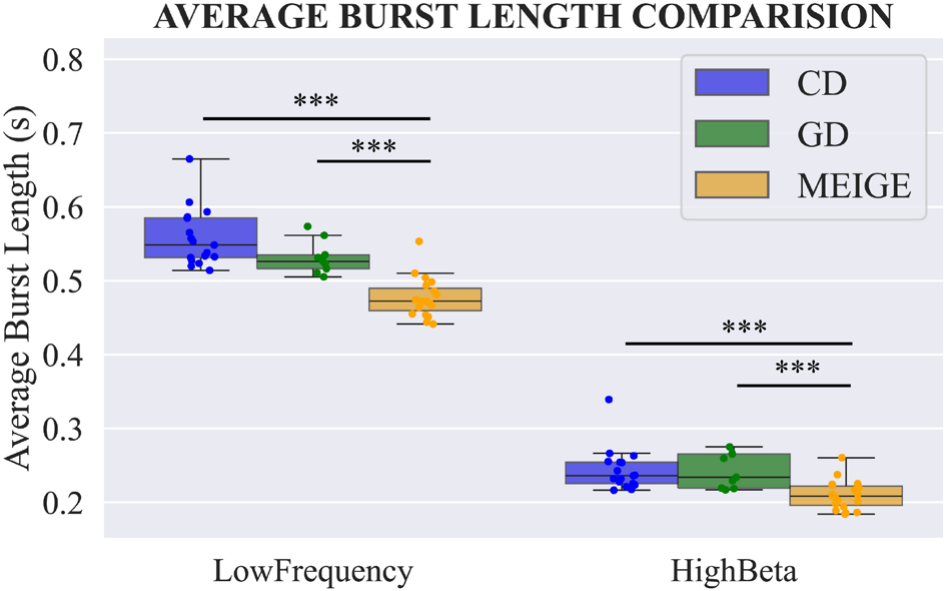
Burst length comparison. CD, Meige, and GD group average burst length comparison revealed the length of low‐frequency and high beta bursts in CD and GD groups is significantly higher than the Meige group. No significant differences were found between CD and GD groups. **p_Bonferroni_ < 0.01, ***p_Bonferroni_ < 0.001, Kruskal–Wallis test. CD, cervical dystonia; GD, generalized dystonia.

We conducted an additional analysis of burst length as a potential dystonic biomarker. The average low‐frequency and high‐beta burst length was longer in CD and GD compared to the Meige group (both *p* < 0.001; Figure 3).

### 3.3 Coherence Analysis and Frequency‐Specific Cortex Interactions

**FIGURE 4:**
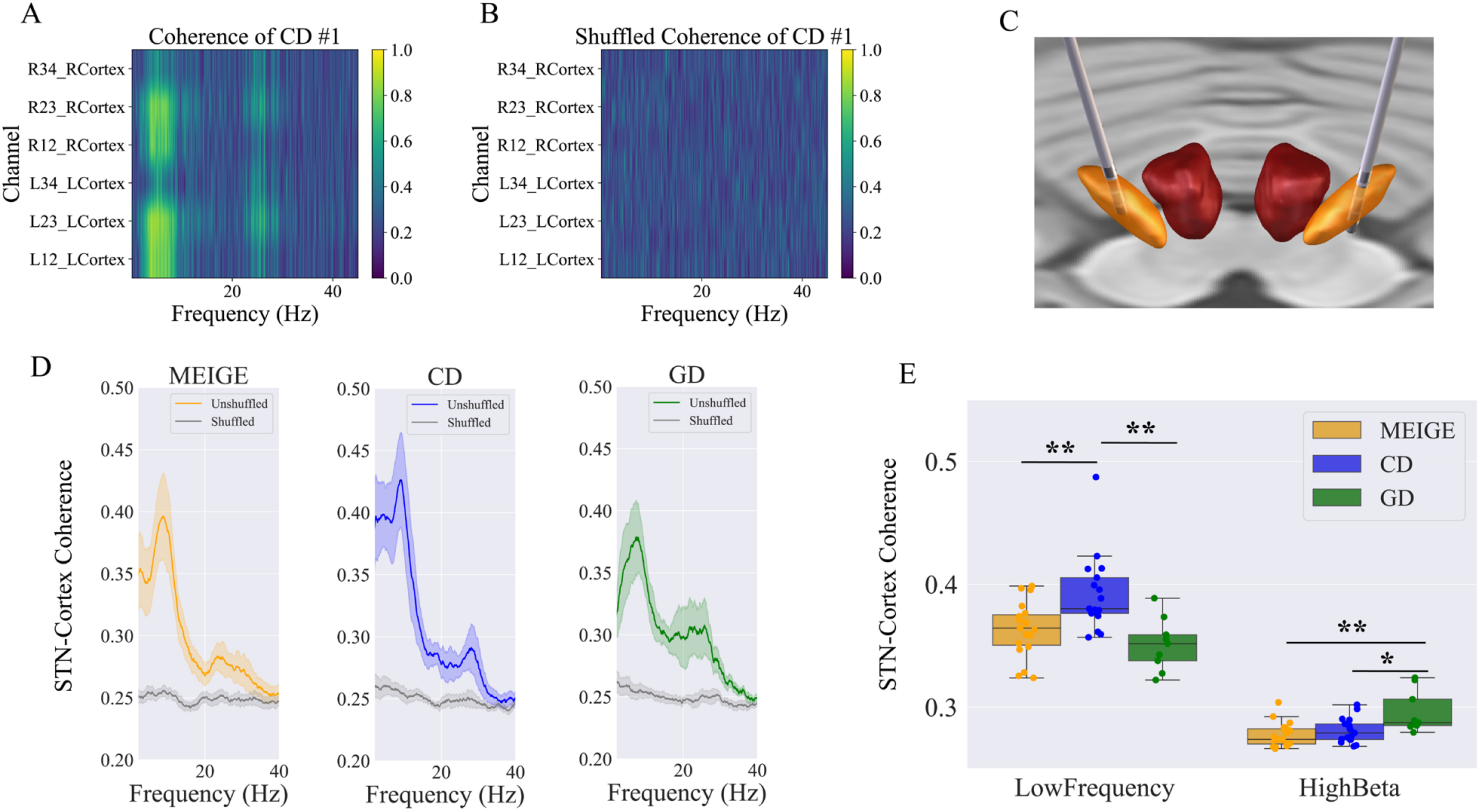
Subthalamic nucleus‐cortex coherence analysis. (A) Example patient CD #1 coherence between STN contacts and ipsilateral F3‐C3 or F4‐C4 EEG channels, showing that low‐frequency coherence with ipsilateral cortex is greatest for L12, L23, R12, and R23, and high beta coherence is greatest for L23 and R23. (B) Phase shuffled surrogate data show little STN‐cortex coherence. (C) DBS lead reconstruction of patient CD1, showing contacts L23 and R23 are located in dorsal. (D) STN‐cortex coherence results show significant low‐frequency and high beta peaks in all dystonia groups (Wilcoxon signed‐rank test) (E) Significant differences in STN‐cortex coherence can be seen for low‐frequency and high beta bands among dystonia groups. The CD group had greater low‐frequency coherence than the GD and Meige groups; the GD group had greater high beta coherence than the Meige and CD groups. *p_Bonferroni_ < 0.05, **p_Bonferroni_ < 0.01, Kruskal–Wallis test. CD, cervical dystonia; GD, generalized dystonia.

Coherence analysis is vital in neural signal analysis for understanding the connectivity and synchronization between brain regions. An example revealed prominent interactions in the low‐frequency and high beta bands between the STN and ipsilateral cortex. Coherence was presented between the STN and ipsilateral cortex, with high beta coupling tending to localize more dorsally within the STN (Figure 4A–C). Examining group‐level patterns, there was significant coherence in both low‐frequency and beta bands across all dystonia subtypes (CD, Meige, and GD) (Figure 4D). The distribution of coherence peaks within these bands showed no significant differences between the groups (*p* > 0.05).

**FIGURE 5:**
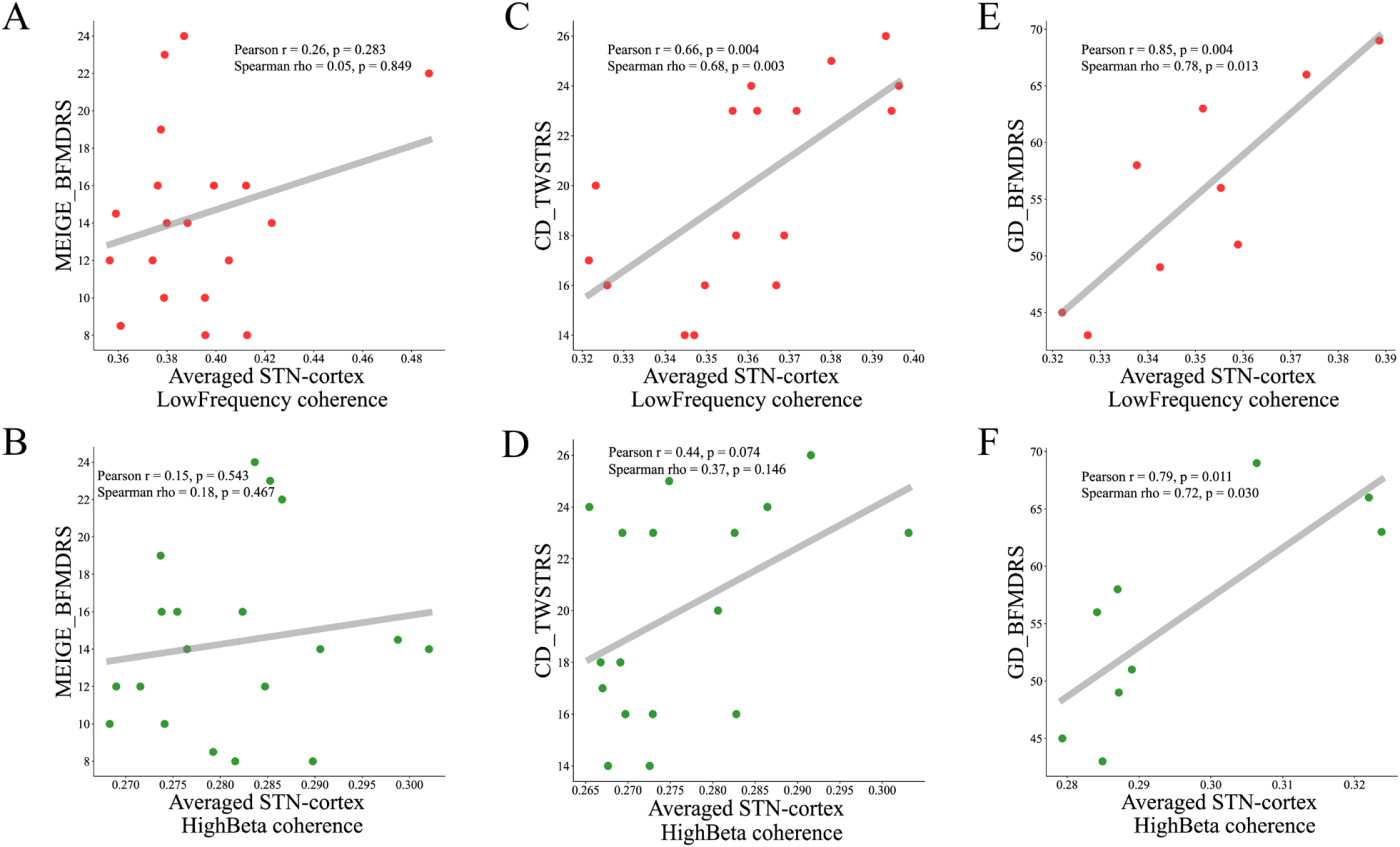
Correlation analysis between STN‐cortex coherence and syndrome severity. (A, B) Meige: No significant correlations were found between either low‐frequency or high‐beta coherence and Meige BFMDRS scores. (C, D) CD: A significant positive correlation was observed between low‐frequency coherence and TWSTRS scores. High‐beta coherence did not show a significant correlation with TWSTRS scores. (E, F) GD: Both low‐frequency and high‐beta coherence displayed significant positive correlations with BFMDRS scores. BFMDRS, Burke‐Fahn‐Marsden Dystonia Rating Scale (motor); CD, cervical dystonia; GD, generalized dystonia; TWSTRS, Toronto Western Spasmodic Torticollis Rating Scale (severity).

Comparison of the low‐frequency and high beta band coherence among the groups showed that the CD group had greater coherence in the low‐frequency band than the Meige and GD groups (*p* < 0.01), while the GD group had greater coherence in the high beta band than the CD (*p* < 0.05) and Meige (*p* < 0.01) groups (Figure 4E). We further analyzed the correlation of coherence values and clinical scores. No significant correlation was found in the Meige group (Figure 5A,B). For the CD group, a significant correlation was found between low‐frequency band coherence and the TWSTRS (severity) scores (rho = 0.68, *p* < 0.01). For the GD group, a significant correlation was found between low‐frequency and high beta band coherence and the BFMDRS (motor) scores (low‐frequency coherence: rho = 0.78, *p* < 0.05; high beta coherence: rho = 0.72, *p* < 0.05) (Figure 5C–F).

### 3.4 Subthalamic Peak Amplitude Analysis

**FIGURE 6:**
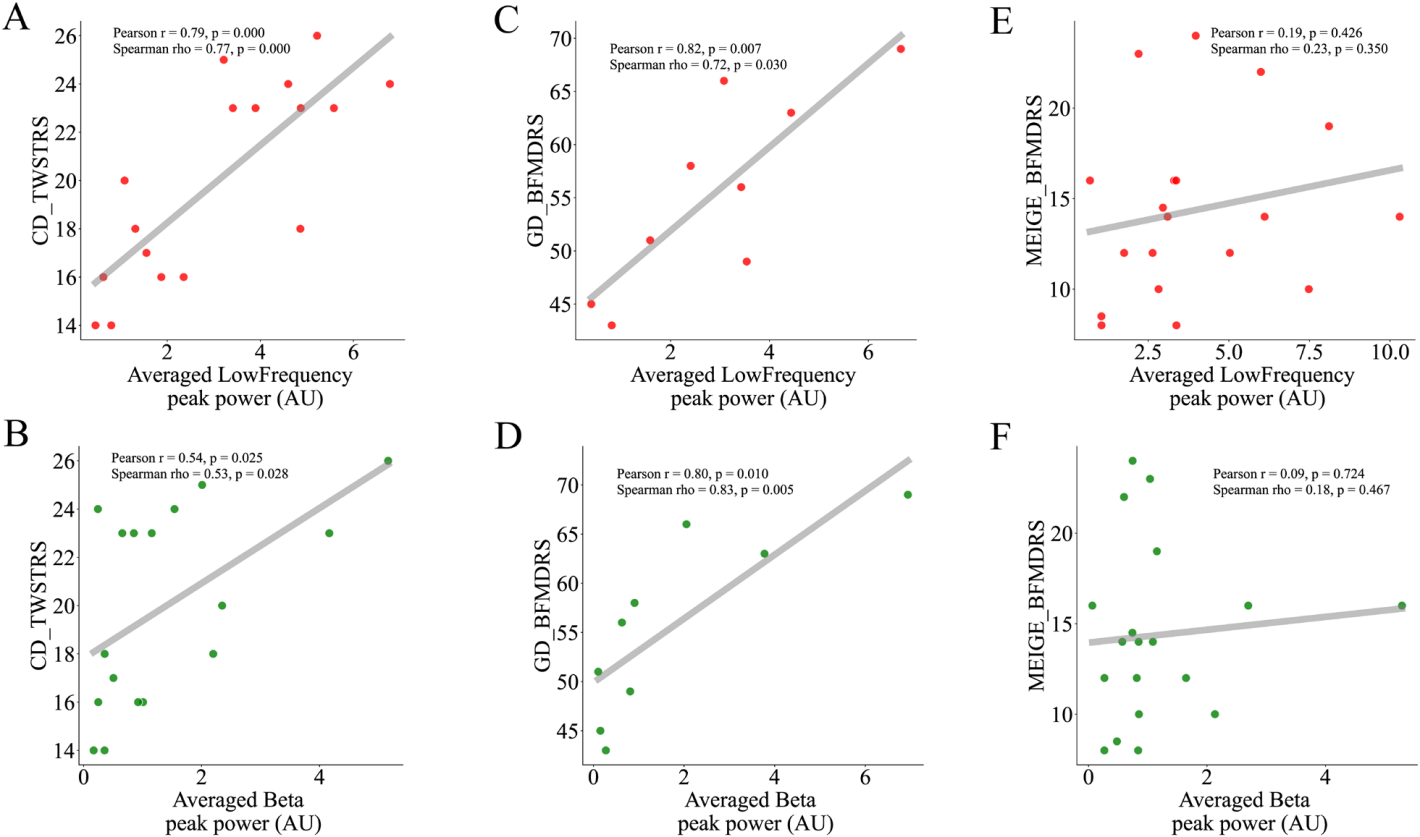
Correlation analysis between peak amplitude and syndrome severity. (A, B) CD: A significant positive correlation was observed between both low‐frequency and beta peak amplitude and TWSTRS scores. (C, D) GD: Both low‐frequency and beta peak amplitude displayed significant positive correlations with BFMDRS scores. (E, F) Meige: No significant correlations were found between either low‐frequency or beta peak amplitude and Meige BFMDRS scores. BFMDRS, Burke‐Fahn‐Marsden Dystonia Rating Scale (motor); CD, cervical dystonia; GD, generalized dystonia; TWSTRS, Toronto Western Spasmodic Torticollis Rating Scale (severity).

The average preoperative TWSTRS (severity) score was 20.00 ± 4.08 in the CD group. The average preoperative BFMDRS (motor) score was 14.37 ± 4.86 in the Meige group, and 55.56 ± 9.25 in the GD group. Given that peak amplitude in GPi has been shown to correlate with dystonic severity in a previous study [4], we investigated the relationship between low‐frequency and beta STN peaks with clinical scores. Subthalamic low‐frequency peak power averaged across all contact pairs in each patient correlated significantly with preoperative TWSTRS (severity) scores in the CD group (rho = 0.77, *p* < 0.001), and with BFMDRS (motor) scores in the GD group (rho = 0.72, *p* < 0.05). For high beta peak power, a significant correlation was found in the CD group with TWSTRS (severity) scores (rho = 0.53, *p* < 0.05), and in the GD group with BFMDRS (motor) scores (rho = 0.83, *p* < 0.01) (Figure 6A–D). Neither low‐frequency nor beta peak power correlated significantly with BFMDRS (motor) scores in the Meige group (low‐frequency: rho = 0.23, *p* > 0.3; beta: rho = 0.18, *p* > 0.4) (Figure 6E,F).

### 3.5 Preoperative Dystonic Symptom Severity Prediction

**FIGURE 7:**
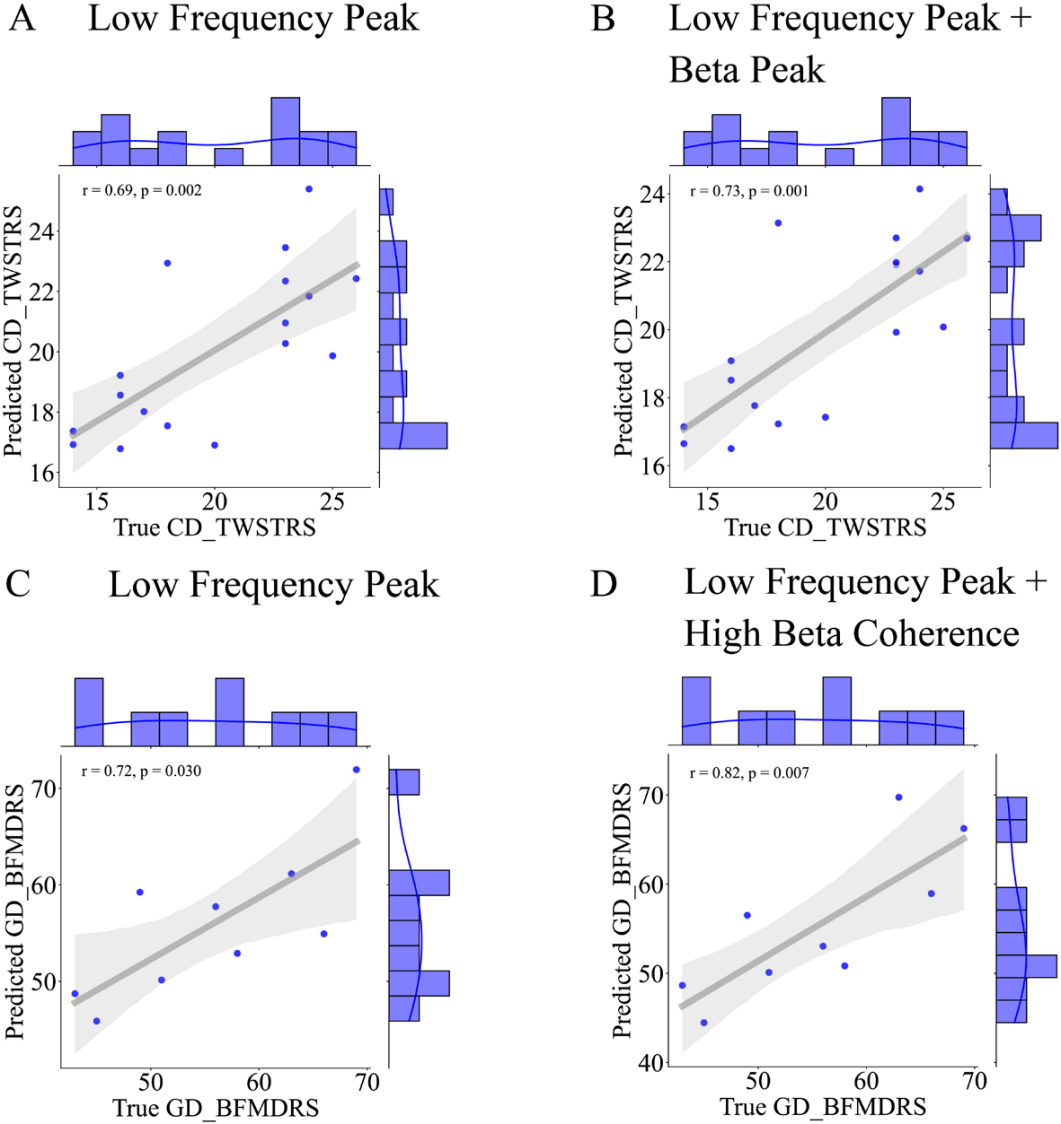
Syndrome severity prediction in CD and GD. (A) To predict TWSTRS scores in the CD group, the linear regression model and leave‐one validation method's performance was 69% (*p* = 0.002) using low‐frequency peak power. (B) Coefficient was elevated to 73% when adding beta peak as another feature (*p* = 0.001). (C) To predict BFMDRS scores in the GD group, the linear regression model and leave‐one validation method's performance was 72% (*p* = 0.030) using low‐frequency peak power. (D) Coefficient was elevated to 82% when adding high beta coherence as another feature (*p* = 0.007). BFMDRS, Burke‐Fahn‐Marsden Dystonia Rating Scale (motor); CD, cervical dystonia; GD, generalized dystonia; TWSTRS, Toronto Western Spasmodic Torticollis Rating Scale (severity).

To further validate this link, we applied a linear regression model and leave‐one‐subject‐out cross validation to quantify the predictive ability of power for symptom severity. Ten power and coupling features were used to build the decoding model. Following the mRMR feature selection, the most important features were: low‐frequency and beta peak power for CD, low‐frequency and low‐frequency peak power and high beta coherence for GD, low‐frequency peak power and low‐frequency coherence for Meige. The model had a predictive performance of 69% in the CD group and 72% in the GD group (Figure 7A,C) using low‐frequency peak power. Adding beta peak power raised the performance to 73% in the CD group. Adding high beta coherence raised the performance to 82% in the GD group (Figure 7B,D). Performance was not good in the Meige group (14%).

## 4 Discussion

Our study demonstrates the presence of distinct oscillatory patterns within the STN across three dystonia types. We observed strong low‐frequency oscillations in the STN. Furthermore, we identified significant high beta activity, with greater prominence in the CD and GD groups than the Meige group. Coherence analysis revealed enhanced cortex‐STN coupling in the low‐frequency and high beta bands, particularly evident in the CD and GD groups. Importantly, this high beta activity was most distributed in STN regions connected to the hyperdirect pathway.

### 4.1 Low‐Frequency Activity and Dystonia

In recent years, the STN has become an increasingly popular target for DBS in the treatment of dystonia, consuming less power and exhibiting a faster onset of action compared to GPi‐DBS in Meige syndrome [18], and showing similar clinical efficacy to GPi‐DBS in CD and GD [19]. Previous research on the GPi‐DBS has demonstrated that low‐frequency oscillations within the GPi are closely related to the pathophysiological mechanisms of dystonia, both in terms of coupling with electromyography and clinical score correlations [4, 20]. Naturally, questions have been raised as to whether similar dystonic biomarkers exist in the STN. This study found that low‐frequency activity exists within the STN, being localized to the sensorimotor region, a finding consistent with previous studies reporting better therapeutic outcomes for sensorimotor STN‐DBS [21]. Additionally, in the CD and GD group, low‐frequency activity correlated with the severity of clinical symptoms. No significant correlation was found in the Meige group, possibly due to the smaller muscle groups involved and its distinct pathophysiology, such as in the disruption of visuo‐motor pathways [22].

The distinction of Meige from CD and GD was further evidenced in the burst analysis, where average low frequency burst length was longer in GD and CD compared to Meige. Previous studies have highlighted the pathological significance of longer bursts, including in Parkinson's disease (PD) where longer beta bursts are associated with rigidity and bradykinesia [23] and in Tourette's syndrome, where the length of low‐frequency bursts correlates positively with the severity of tics [24]. Despite this, we cannot exclude the possibility that the BFMDRS for Meige syndrome evaluation lacks sufficient sensitivity to accurately capture the full spectrum of the syndrome's manifestations, as no significant correlation was found between low‐frequency activity and syndrome severity.

Crucial to the motor functions disrupted in dystonia is the cortex‐basal ganglia circuitry. Previous research suggests that dystonia involves changes in cortical plasticity, a view supported by certain types of dystonia related to repetitive behaviors, such as musician's dystonia [25]. Our findings of prominent cortex‐STN functional coupling, particularly in the low‐frequency band, support the hypothesis that low‐frequency activity in dystonia acts as a circuit disruptor, potentially disrupting healthy information transmission to produce repetitive and enhanced activities that should otherwise cease [26, 27]. Indeed, our findings of stronger low‐frequency coupling in CD are one potential distinct mechanism of this dystonia type. A recent imaging comparison of the best target for GPi‐DBS in CD and GD revealed that optimal outcomes in CD were achieved by targeting the pallidosubthalamic pathway, whereas modulating pallidothalamic fibers was more effective for GD [28]. The pallidosubthalamic pathway is associated with the indirect pathway, utilizing the substantia nigra as the output structure, while the pallidothalamic pathway primarily involves the GPi as the output structure [28]. This distinction may explain the stronger low‐frequency circuit resonance seen in CD, leading to greater cortex‐STN coupling in the low‐frequency range.

### 4.2 High Beta Activity and Dystonia

Beta oscillations have been widely studied in Parkinson's disease (PD), where low beta activity is pathologically elevated and associated with rigidity and bradykinesia [29]. In contrast, our study highlights that high beta activity is the more prominent feature in dystonia STN‐LFPs, with most peaks falling within the high beta range, distinguishing it from the beta‐band abnormalities commonly observed in PD. A previous study reported no differences in beta band activity between PD and dystonia [29]. Our study builds on this research, emphasizing the greater relevance of the high‐beta frequency band in dystonia.

Recent studies have shown that low and high beta frequencies have different pathophysiological implications, with low beta involved primarily in motor regulation, and high beta more closely related to hyperdirect pathway activity [30, 31, 32]. Interestingly, a PD computational modeling study suggested that the pathological synchrony was induced by the hyperdirect pathway [30, 33, 34]. In our study, high beta activity is most pronounced in the dorsal STN, a region associated with the hyperdirect pathway. Further evidence comes from the cortex‐STN coupling results, where the highest coupling was observed with the dorsal STN. Moreover, high beta cortex‐STN coupling in GD exceeded that observed in CD. This idea is also supported by the earlier imaging study, where modulation of corticofugal tracts from the somatomotor head and neck region was associated with optimal outcomes in CD, while tracts from the whole somatotopical domain were associated with GD [28].

In CD and GD, symptom severity was positively correlated with beta peak power and cortex‐STN high beta coupling strength, whereas no significant correlations were found for Meige syndrome, which might be due to the differential pathophysiology of Meige syndrome.

How low and high beta oscillations are regulated during movement is crucial for fully understanding their pathophysiological significance. However, our dataset lacks movement‐related tasks to explore this. Future studies should focus on beta dynamics during movement in dystonia and their comparison with Parkinson's disease.

### 4.3 Closed‐Loop Biomarkers for Treating Dystonia With STN‐DBS

The development of closed‐loop electrical stimulation is a promising advancement for DBS therapy, with the potential to enhance therapeutic efficacy while reducing side effects [35]. In PD, enhanced low beta oscillations have been established as a biomarker of rigidity and bradykinesia, with DBS targeting the suppression of these low beta oscillations associated with clinical improvement [36]. Similarly, closed‐loop stimulation in dystonia using oscillatory biomarkers may be highly effective [37]. Previous studies in the GPi have demonstrated the widespread presence of low‐frequency activity and pathological synchrony in circuits, laying the groundwork for closed‐loop stimulation development for dystonia [13, 38]. Some previous studies have indeed found increased low‐frequency power in PD patients with dyskinesia, suggesting that low frequency oscillations may reflect a common biomarker for hyperkinetic involuntary movements [39]. However, different symptoms may have distinct underlying pathological mechanisms, so combining other frequency bands is necessary for better detection. Recent studies have found that narrowband gamma is more strongly associated with dyskinesia and is a potential better biomarker [40]. Our study furthers previous work, showing that both low‐frequency oscillations and high beta activity in the STN are correlated with clinical symptoms of dystonia. Interestingly, combining additional features provided a better prediction of clinical symptom severity than using low‐frequency oscillations alone, a finding also seen in a Tourette's syndrome severity prediction study [24]. Our research offers valuable insights for improving STN‐DBS therapy in dystonia patients, and we note two key applications. First, closed‐loop DBS systems could be significantly enhanced by incorporating multiple features apart from low‐frequency activity, such as high beta and low gamma activity. By using a more comprehensive picture of brain activity, stimulation can be better personalized to target the specific needs of each patient. Second, the identification of distinct oscillatory patterns across dystonia subtypes opens doors for the development of subtype‐specific biomarkers. Since different dystonia types likely involve unique underlying mechanisms, the most effective biomarkers for stimulation adjustments may also vary.

## 5 Limitations

Our study has several limitations. First, we did not perform EMG analysis, which limits our ability to correlate brain activity with muscle activity. Furthermore, capturing muscle activity in Meige syndrome patients is particularly challenging. However, previous research has established a link between low‐frequency power and muscle coherence [4, 41]. Therefore, we employed pre‐operative symptom evaluation by an experienced neurologist, a standard approach in similar biomarker studies. It is important to emphasize that this study aimed to identify and characterize STN activity patterns associated with different dystonia types, not to predict real‐time muscle contractions. Second, microlesion effects from electrode implantation could influence symptoms and basal ganglia activity. While long‐term recordings using sensing devices are crucial for long‐term validation, the persistence of symptoms during recordings mitigates this concern to some extent. Third, dystonia itself can introduce artifacts, particularly in the low‐frequency range susceptible to movement contamination. To address this, we rigorously excluded noisy data segments and contact pairs as we did a long‐time recording, and focused on clean periods for analysis. Additionally, the distinct distributions of low and high beta power within the STN further suggest that movement artifacts are unlikely to be a major confounding factor. Our fourth limitation is that we only used subjective rating scales. In the future, AI tools could be utilized for more objective assessments.

## 6 Conclusion

Our study identified the low‐frequency and high beta activity within the STN in dystonia. These activities showed unique spatial distributions within the STN and were correlated with symptom severity, suggesting a close relationship to the pathophysiological mechanisms of dystonia.

## Author Contributions

Zhu Guan‐Yu designed the experiment, analyzed and interpreted the data, and drafted the manuscript. Yin Zi‐Xiao collected the LFPs data and analyzed the data; Chen Ying‐Chuan collected the LFPs data. Thomas Binns drafted the manuscript. Timon Merk analyzed and interpreted the data. Ma Ruo‐Yu, Du Ting‐Ting, Liu Yu‐Ye, and Xie Hu‐Tao collected the clinical data. Shi Lin, Yang An‐Chao, and Meng Fan‐Gang helped supervise the data analysis. Wolf‐Julian Neumann, Andrea A. Kühn, and Zhang Jian‐Guo supervised the design of the experiment and data analysis and revised the manuscript. All authors approved the final version of the manuscript.

## Conflicts of Interest

The authors declare no conflicts of interest.

## Supporting information


Table S1.

## Data Availability

The datasets used and analyzed during the current study are available from the corresponding author on reasonable request.
