# Supplementary material for: Subthalamic Nucleus Oscillatory Characteristics in Meige, Cervical Dystonia and Generalized Dystonia
Source: Ann Clin Transl Neurol. 2025 Apr 3;12(5):1082–92. doi: 10.1002/acn3.70040 (PMC12093343; doi:10.1002/acn3.70040)
Supplement: Supplementary file 1 — Table S1. [file ACN3-12-1082-s001.docx]

# SUPPLEMENTARY TABLE

| **Supplementary Table 1. Clinical summary** | | | | | |
| --- | --- | --- | --- | --- | --- |
| **Group** | **Age at onset(yrs)/Sex** | **Duration**  **(yrs)** | **Affected body part** | **Previous**  **treatment** | **Clinical**  **evaluation** |
| MEIGE | 62/Male | 3 | Eye, Month | Baclofen,Botulinum | BFMDRS-M:14 |
| MEIGE | 65/Male | 9 | Eye, Month, Speech | Baclofen,Botulinum | BFMDRS-M:24 |
| MEIGE | 45/Female | 3 | Eye, Month | Botulinum | BFMDRS-M:14 |
| MEIGE | 50/Female | 13 | Eye, Month | Baclofen, Trihexyphenidyl,Botulinum | BFMDRS-M:8 |
| MEIGE | 60/Female | 2 | Eye, Month | Botulinum | BFMDRS-M:8.5 |
| MEIGE | 51/Male | 2 | Eye, Month | Botulinum, Baclofen, Clonazepam | BFMDRS-M:16 |
| MEIGE | 42/Male | 5 | Eye, Month | Botulinum | BFMDRS-M:8 |
| MEIGE | 69/Female | 1 | Eye, Month | Clonazepam,Botulinum | BFMDRS-M:12 |
| MEIGE | 64/Male | 1 | Eye, Month | Clonazepam,Botulinum | BFMDRS-M:14.5 |
| MEIGE | 54/Female | 4 | Eye, Month | Baclofen,Diazepam | BFMDRS-M:10 |
| MEIGE | 50/Female | 7 | Eye, Month, Speech | N.A. | BFMDRS-M:22 |
| MEIGE | 41/Male | 4 | Eye, Month | Baclofen,Clonazepam,Botulinum | BFMDRS-M:12 |
| MEIGE | 51/Male | 5 | Eye, Month | Botulinum | BFMDRS-M:10 |
| MEIGE | 66/Male | 4 | Eye, Month | Trihexyphenidyl,Botulinum | BFMDRS-M:14 |
| MEIGE | 61/Female | 1 | Eye, Month | Botulinum,Diazepam | BFMDRS-M:12 |
| MEIGE | 44/Female | 3 | Eye, Month, Speech | Botulinum | BFMDRS-M:23 |
| MEIGE | 53/Male | 4 | Eye, Month, Speech | Botulinum | BFMDRS-M:19 |
| MEIGE | 64/Female | 6 | Eye, Month | Baclofen,Clonazepam,Botulinum | BFMDRS-M:16 |
| MEIGE | 60/Male | 4 | Eye, Month | Botulinum | BFMDRS-M:16 |
| CD | 42/Female | 5 | Neck | Botulinum,Clonazepam | TWSTRS:23 |
| CD | 58/Female | 8 | Neck | Trihexyphenidyl,Botulinum | TWSTRS:16 |
| CD | 65/Male | 7 | Neck | Botulinum,Trihexyphenidyl | TWSTRS:23 |
| CD | 40/Male | 3 | Neck | Botulinum,Baclofen | TWSTRS:24 |
| CD | 33/Female | 2 | Neck | Botulinum, Baclofen, Clonazepam | TWSTRS:14 |
| CD | 64/Female | 20 | Neck | N.A. | TWSTRS:23 |
| CD | 53/Male | 8 | Neck | Botulinum | TWSTRS:26 |
| CD | 49/Female | 10 | Neck | Botulinum, Baclofen, | TWSTRS:24 |
| CD | 54/Female | 15 | Neck | Botulinum,Clonazepam,Baclofen | TWSTRS:20 |
| CD | 47/Male | 9 | Neck | Botulinum, Baclofen, | TWSTRS:16 |
| CD | 51/Female | 9 | Neck | Clonazepam,Baclofen | TWSTRS:18 |
| CD | 39/Male | 4 | Neck | Baclofen, Trihexyphenidyl,Botulinum | TWSTRS:14 |
| CD | 55/Female | 3 | Neck | Botulinum | TWSTRS:16 |
| CD | 52/Female | 11 | Neck | Botulinum,Baclofen | TWSTRS:23 |
| CD | 47/Male | 16 | Neck | N.A. | TWSTRS:25 |
| CD | 64/Female | 12 | Neck | Botulinum | TWSTRS:17 |
| CD | 65/Male | 5 | Neck | Botulinum,Trihexyphenidyl | TWSTRS:18 |
| GD | 13/Male | 4 | Month, Speech, Neck, Trunk, Limb | Trihexyphenidyl,Clonazepam | BFMDRS-M:63 |
| GD | 33/Female | 10 | Trunk, Limb | Baclofen, Trihexyphenidyl,Clonazepam | BFMDRS-M:45 |
| GD | 8/Male | 1 | Eye, Month, Neck, Trunk, Limb | Baclofen, Clonazepam | BFMDRS-M:66 |
| GD | 67/Male | 4 | Eye, Month, Limb | Baclofen, Trihexyphenidyl,Clonazepam | BFMDRS-M:58 |
| GD | 28/Female | 3 | Trunk, Limb | Levedopa, Baclofen | BFMDRS-M:43 |
| GD | 27/Male | 6 | Neck, Trunk, Limb | Botulinum, Baclofen, Clonazepam | BFMDRS-M:56 |
| GD | 32/Male | 2 | Trunk, Limb | Tetrabenazine, Clonazepam | BFMDRS-M:49 |
| GD | 14/Male | 12 | Eye, Month, Speech, Neck, Trunk, Limb | Baclofen | BFMDRS-M:69 |
| GD | 36/Female | 20 | Neck, Trunk, Limb | Botulinum, Baclofen | BFMDRS-M:51 |
| M = male; F = female; BFMDRS-M = Burke-Fahn-Marsden dystonia rating scale-Motor part; TWSTRS-Toronto Western Spasmodic Torticollis Rating Scale (Severity part). | | | | | |
